# Draft Genome Sequence of Rhodovulum sp. Strain NI22, a Naphthalene-Degrading Marine Bacterium

**DOI:** 10.1128/genomeA.01475-14

**Published:** 2015-01-22

**Authors:** Lisa M. Brown, Thusitha S. Gunasekera, Loryn L. Bowen, Oscar N. Ruiz

**Affiliations:** a University of Dayton Research Institute, Dayton, Ohio, USA; b Air Force Research Laboratory, Aerospace Systems Directorate, Fuels and Energy Branch, Wright-Patterson AFB, Ohio, USA

## Abstract

Rhodovulum sp. strain NI22 is a hydrocarbon-degrading member of the genus Rhodovulum. The draft genome of Rhodovulum sp. NI22 is 3.8 Mb in size, with 3,756 coding sequences and 64.4% G+C content. The catechol and gentisate pathways for naphthalene degradation are predicted to be present in Rhodovulum sp. NI22.

## GENOME ANNOUNCEMENT

Rhodovulum sp. strain NI22 is an isolate of the genus Rhodovulum, capable of degrading hydrocarbons aerobically. This bacterium was isolated from coastal seawater from Key West, Florida, USA, after having been exposed to JP-5 aviation fuel.

Rhodovulum sp. NI22 is a Gram-negative, mesophilic, slightly halophilic, chemo-organotrophic, rod-shaped bacterium that can use naphthalene as its sole carbon and energy source. Based on the 16S rRNA gene sequence, Rhodovulum sp. NI22 is 95.0% similar to Rhodovulum marinum. Members of the genus Rhodovulum are often found in hydrocarbon-polluted marine environments, and some have been reported to degrade aromatic compounds such as phthalate (1–3).

Rhodovulum sp. NI22 was confirmed to efficiently degrade naphthalene in jet and diesel fuels. Therefore, we have sequenced its genome, to understand the underlying naphthalene degradation mechanisms of this bacterium.

Rhodovulum sp. NI22 was sequenced on a Roche 454-GS Junior platform using a whole-genome shotgun approach, resulting in 346,448 reads. The sequence reads were assembled with the Roche *de novo* Assembly software. The assembly reported 120 large (>500 bp) contigs, with an *N*_50_ of 57,970 bp. The longest contig was 288,171 bp. The draft genome sequence was 3,819,905 bases in length, with a G+C content of 64.4%. Rapid genome annotation using the RAST annotation server (4) described 3,756 coding sequences (CDSs) and 45 structural RNAs, which consisted of one 16S rRNA, one 23S rRNA, and 43 tRNAs. The coding sequences were classified into 442 subsystems, of which amino acids and derivatives (*n* = 370 CDSs); carbohydrates (*n* = 357); cofactors, vitamins, prosthetic groups, and pigments (*n* = 281); protein metabolisms (*n* = 255); membrane transport (*n* = 174); fatty acids, lipids, and isoprenoids (*n* = 144); stress response (*n* = 135); RNA metabolism (*n* = 134); nucleosides and nucleotides (*n* = 109); cell wall and capsule (*n* = 97); and metabolisms of aromatic compounds (*n* = 70) were the most abundant subsystems.

The NCBI Prokaryotic Genome Annotation Pipeline (http://www.ncbi.nlm.nih.gov/genome/annotation_prok) predicted naphthalene 1,2-dioxygenase (*ndo*), catechol 1,2-dioxygenase (*catA*), and gentisate 1,2-dioxygenase (*gdo*) genes to be present in the genome of Rhodovulum sp. NI22. Naphthalene 1,2-dioxygenase undergoes the dihydroxylation of naphthalene during the first step of the upper catabolic pathway, while catechol 1,2-dioxygenase and gentisate 1,2-dioxygenase further metabolize salicylate via the catechol or gentisate lower pathways, respectively (5–7). Other important hydrocarbon-degrading genes observed were benzene 1,2-dioxygenase, biphenyl 2,3-dioxygenase, 2-nitropropane dioxygenase, protocatechuate 3,4-dioxygenase, and alkane 1-monooxygenase (*alkB*). The study of Rhodovulum sp. NI22 genome will provide valuable insight into the metabolism and adaptation of marine bacteria to aromatic hydrocarbon compounds.

### Nucleotide sequence accession number.

This whole-genome shotgun project has been deposited in DDBJ/EMBL/GenBank under the accession number JQFU00000000.

## References

[1] Liu Z, Liu J 2013 Evaluating bacterial community structures in oil collected from the sea surface and sediment in the northern Gulf of Mexico after the *Deepwater Horizon* oil spill. Microbiol Open 2:492–504. doi:10.1002/mbo3.89. PMC3684762 23568850

[2] Iwaki H, Nishimura A, Hasegawa Y 2012 Isolation and characterization of marine bacteria capable of utilizing phthalate. World J Microbiol Biotechnol 28:1321–1325. doi:10.1007/s11274-011-0925-x. 22805854

[3] Teramoto M, Suzuki M, Hatmanti A, Harayama S 2010 The potential of *Cycloclasticus* and *Altererythrobacter* strains for use in bioremediation of petroleum-aromatic-contaminated tropical marine environments. J Biosci Bioeng 110:48–52. doi:10.1016/j.jbiosc.2009.12.008. 20541115

[4] Aziz RK, Bartels D, Best AA, DeJongh M, Disz T, Edwards RA, Formsma K, Gerdes S, Glass EM, Kubal M, Meyer F, Olsen GJ, Olson R, Osterman AL, Overbeek RA, McNeil LK, Paarmann D, Paczian T, Parrello B, Pusch GD, Reich C, Stevens R, Vassieva O, Vonstein V, Wilke A, Zagnitko O 2008 The RAST server: Rapid Annotations using Subsystems Technology. BMC Genomics 9:75. doi:10.1186/1471-2164-9-75. 18261238 PMC2265698

[5] Simon MJ, Osslund TD, Saunders R, Ensley BD, Suggs S, Harcourt A, Suen WC, Cruden DL, Gibson DT, Zylstra GJ 1993 Sequences of genes encoding naphthalene dioxygenase in *Pseudomonas putida* strains G7 and NCIB 9816–4. Gene 127:31–37. doi:10.1016/0378-1119(93)90613-8. 8486285

[6] Tomás-Gallardo L, Gómez-Álvarez H, Santero E, Floriano B 2014 Combination of degradation pathways for naphthalene utilization in *Rhodococcus* sp. strain TFB. Microb Biotechnol 7:100–113. doi:10.1111/1751-7915.12096. 24325207 PMC3937715

[7] Feng Y, Khoo HE, Poh CL 1999 Purification and characterization of gentisate 1,2-dioxygenases from *Pseudomonas alcaligenes* NCBI 9867 and *Pseudomonas putida* NCBI 9869 and *Pseudomonas putida* NCIB 9869. Appl Environ Microbiol 65:946–950. 10049846 10.1128/aem.65.3.946-950.1999 PMC91127

